# Network Pharmacology and Molecular Docking Validation to Reveal the Pharmacological Mechanisms of Kangai Injection against Colorectal Cancer

**DOI:** 10.1155/2022/3008842

**Published:** 2022-08-21

**Authors:** Bo-Bo Zheng, Quan Wang, Yumin Yue, Jiang Li, Xiao-Jun Li, Xin Wang

**Affiliations:** ^1^Department of General Surgery, Shaanxi Provincial People's Hospital, Xi'an, Shaanxi, China; ^2^Laboratory of Surgical Oncology, Peking University People's Hospital, Beijing, China; ^3^National Cancer Center/Cancer Hospital, Chinese Academy of Medical Sciences and Peking Union Medical College, Beijing, China; ^4^Department of Gastroenterology, First Affiliated Hospital of Xi'an Jiaotong University, Xi'an, Shaanxi Province, China

## Abstract

**Background:**

Kangai injection is a traditional Chinese medicine (TCM) mixed by extracts from astragalus, ginseng, and kurorinone with modern technology. It is a commonly used antitumor injection in China, but the mechanism of Kangai injection in the treatment of colorectal cancer (CRC) is still unclear. The purpose of this study is to explore the mechanism of Kangai injection against CRC using network pharmacology and molecular docking technology.

**Methods:**

Targets of Kangai injection in CRC were predicted by SwissTargetPrediction and DisGeNET databases. Gene Ontology (GO) analysis and Kyoto Encyclopedia of Genes and Genomes (KEGG) were performed by using the DAVID database. A component-disease-target gene-pathway network was constructed by Cytoscape 3.8.0 software.

**Results:**

114 overlapping targets of Kangai injection and CRC were used to construct a PPI network, and the top 10 hub targets of Kangai injection were rated from high to low as *TP53*, *VEGFA*, *EGFR*, *TNF*, *ESR1*, *STAT3*, *HSP90AA1*, *HDAC1*, *AR*, and *MMP9*. The ingredient-target-disease interactive network was constructed, which included 22 compounds and 114 overlapping targets with 161 nodes and 707 edges. Entries of enrichment analysis were obtained based on *P* value (<0.05), which included 19 of GO-MF, 217 of GO-BP, 8 of GO-CC, and 13 KEGG. Molecular docking analysis showed that Kangai injection strongly interacted with top 10 hub target proteins.

**Conclusion:**

Network pharmacology intuitively showed the multicomponent, multiple targets, and multiple pathways of Kangai injection in the treatment of CRC. The molecular docking experiment verified that compounds of Kangai injection had good binding ability with top 10 hub target proteins as well.

## 1. Introduction

On a global scale, colorectal cancer (CRC) is the third most commonly diagnosed cancer (6.1% of the total cancer cases) in both sexes combined and the second leading cause of cancer-related death (9.2% of the total cases) [1].

Traditional Chinese medicine (TCM) has a long history in the treatment of cancer. As early as 3,500 years ago, the word “tumor” was recorded in oracle bone inscriptions [2]. About two thousand years ago, the *The Yellow Emperor's Inner Canon*, a classic book of TCM, discussed the treatment of cancer. In recent decades, TCM has attracted more and more attention in the global medical field. More and more studies have demonstrated the clinical effects of TCM in the treatment of cancer, such as significantly reducing chemotherapy-related toxic and side effects, reducing tumor recurrence and metastasis, and improving the overall survival rate [3–10].

Kangai injection is a TCM mixed by extracts from astragalus, ginseng, and kurorinone with modern technology. Many studies have shown that Kangai injection, as an adjunct to chemotherapy, can enhance the immunity of CRC patients, improve the sensitivity of chemotherapy, reduce the side effects of chemotherapy, and improve the overall survival time [11–16]. A meta-analysis of Kangai injection as a chemotherapy supplement for CRC was published, which included 28 studies involving 2,310 CRC patients and reached similar conclusions [17]. However, the mechanism of Kangai injection in the treatment of CRC is still unclear.

Network pharmacology is the perfect combination of bioinformatics and pharmacology, which is a new field to explore new drug targets [18]. This approach has been widely used since Hopkins first proposed the concept of network pharmacology in 2008 [19]. It is well known that cancer is a polygenic disease and single-target therapy is often not effective in treating cancer. Network pharmacology is to transform the traditional “single drug-single target treatment” into multidrug component-multitarget treatment” concept [20, 21]. Compared with time-consuming laboratory experiments, network pharmacology is more suitable for exploring active ingredients of drugs and therapeutic targets of disease and molecular mechanisms between drugs and disease [22]. With the development of network pharmacology in the field of TCM, the empirical treatment model of TCM is expected to evolve into evidence-based treatment [23].

Network pharmacology is an important recent development in pharmacology. It is a tool which can systematically and deeply explore complexed biological processes and interrelationships [22]. In cancer and other diseases, network pharmacology relies on a multiomics approach to explore the pathological mechanisms of disease and the potential mechanisms of drug therapy at the cellular and molecular levels [24]. TCM has been gradually accepted as a treatment and widely used in all stages of disease across the world. The World Health Organization has been supporting clinical researches of TCM in the treatment of various diseases [25]. The application of network pharmacology in TCM has built a bridge between modern medicine and traditional medicine [26].

In the present study, we performed a network pharmacology analysis of the targets in CRC and Kangai injection, aiming to explore the molecular mechanisms and pathways of the therapeutic effect of Kangai injection. Furthermore, the molecular docking approach was performed to verify the strength of chemical force between Kangai injection and its predicted targets.

## 2. Materials and Methods

### 2.1. Composition of Kangai Injection

The acquisition of Kangai injection components was mainly through the retrieval of the Traditional Chinese Medicine Systems Pharmacology database (TCMSP) (http://tcmspw.com/tcmsp.php) and literature database. Firstly, the MeSH word “Kangai” is input into the search box for retrieval in the TCMSP database. To select the active compounds of Kangai injection, the criterion of oral bioavailability (OB) and drug-likeness (DL) were set to 30% and 0.1, respectively. Then, all studies on Kangai injection in cancer treatment published until September 2020 were screened from both PubMed and China National Knowledge Infrastructure (CNKI, https://www.cnki.net/). The following medical subject heading terms were used: Kang ai or Kangai or Kang'ai or Kang-ai.

### 2.2. Putative Targets of Kangai Injection and Colorectal Cancer

The 3D or 2D structures of all components of Kangai injection were obtained from PubChem (https://pubchem.ncbi.nlm.nih.gov/). The molecular structure of some components is drawn using an online tool in the SwissTargetPrediction (http://www.swisstargetprediction.ch/) or SuperPred (http://prediction.charite.de/index.php?site=chemdoodle_search_target) when it is not readily available.

In order to get as many potential target proteins of Kangai injection as possible, the target proteins were predicted by the 2D structure of all components of Kangai injection using SwissTargetPrediction and SuperPred. “Homo sapiens” was selected for target prediction. Genes related to CRC were acquired from GeneCards (https://www.genecards.org/), Online Mendelian Inheritance in Man (OMIM, https://www.omim.org/), and DisGeNET (https://www.disgenet.org/, version 7.0) by searching with the key words “colorectal cancer”, “colon cancer”, and “rectal cancer”.

### 2.3. Protein-Protein Interaction

Protein-protein interaction (PPI) was used to explore hub genes of CRC and/or Kangai injection components. Each interaction between input proteins was scored by STRING (http://string-db.org/cgi/input.pl), an online PPI database that is widely used in bioinformatics and network pharmacology and can provide all PPI network data for free [27]. The higher the score was, the more reliable the data would be. After clicking “Search” on the first page of the STRING database, the target proteins were entered in the multiple protein retrieval box. Species was limited to Homo sapiens. The top 10 interacting proteins with the highest scores were selected for PPI network construction using Cytoscape 3.8.0 software.

### 2.4. Gene Ontology and Pathway Analysis

Gene Ontology (GO) and Kyoto Encyclopedia of Genes and Genomes (KEGG) enrichment were analyzed by using Database for Annotation, Visualization and Integrated Discovery (DAVID version 6.8, https://david.ncifcrf.gov/) [28], an online database mining the biological behavior and potential pathways of target genes. The visualization of enrichment analysis was realized by the ImageGP website (http://www.ehbio.com/ImageGP).

### 2.5. Network Construction and Analysis

In order to clearly and intuitively understand the mechanism of Kangai injection in the treatment of CRC, we constructed the component-target gene network and component-disease-target gene-pathway network, respectively. All visual networks were generated by Cytoscape 3.8.0 software (http://www.cytoscape.org/) [29].

### 2.6. Molecular Docking

The 2D structure of the Kangai injection components was obtained through the PubChem website. Crystal structure of target proteins was obtained from the Protein Data Bank database (http://www1.rcsb.org/). The specific process of molecular docking included dehydrating all proteins, extracting the original ligands and storing them separately, and finally carrying out molecular docking by AutoDockTools-1.5.6. The smaller the binding energy was, the tighter the binding would be. PyMOL software was used to perform the visualization of components and protein molecular docking.

## 3. Results

### 3.1. Kangai Injection-Related Compounds and Potential Targets

No ingredients of Kangai injection were found from the TCMSP database, so compounds of Kangai injection were screened based on published literatures. A total of 5 studies [14, 30–33] containing information of components of Kangai injection were retrieved, from which 22 active components of Kangai injection were screened (Table 1). The 2D and 3D chemical structures of all the compounds were obtained from the PubChem database. A total of 575 target genes of Kangai injection were obtained from SwissTargetPrediction and SuperPred databases.

**Figure 1:**
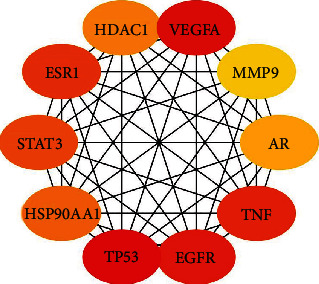
The top 10 hub target genes of Kangai injection.

After removing the duplicates, 160 target genes were left for further analysis. The PPI network of all target genes was constructed to look for hub genes using the Cytoscape software. The top 10 hub genes included *TP53* (degree = 67), *VEGFA* (degree = 57), *EGFR* (degree = 54), *TNF* (degree = 48), *ESR1* (degree = 47), *STAT3* (degree = 44), *HSP90AA1* (degree = 40), *HDAC1* (degree = 37), *AR* (degree = 36), and *MMP9* (degree = 32) (Figure 1).

### 3.2. Enrichment Analysis of Kangai Injection

A total of 160 human genes were identified as target genes of compounds of Kangai injection, and GO and KEGG enrichment analyses were conducted. As shown in Table S1, in regard to molecular functions (MF), higher enrichment was found in carbonate dehydratase activity (*P* = 1.30*E* − 18, FDR = 1.84*E* − 15), amine receptor activity (*P* = 1.98*E* − 14, FDR = 2.80*E* − 11), and hydrolyase activity (*P* = 5.13*E* − 14, FDR = 7.28*E* − 11). The main Kangai injection-related terms in cellular components (CC) contained the plasma membrane part (*P* = 6.96*E* − 11, FDR = 9.10*E* − 08), integral to plasma membrane (*P* = 1.95*E* − 09, FDR = 2.56*E* − 06), and insoluble fraction (*P* = 3.62*E* − 09, FDR = 4.74*E* − 06) (Table S2). Response to organic substance (*P* = 4.99*E* − 19, FDR = 8.60*E* − 16), response to drug (*P* = 9.38*E* − 18, FDR = 1.62*E* − 14), and response to alkaloid (*P* = 4.09*E* − 16, FDR = 7.66*E* − 13) were most closely related to biological processes (BP) (Table S3). KEGG enrichment analysis showed that “nitrogen metabolism,” “steroid hormone biosynthesis,” “androgen and estrogen metabolism,” “Notch signaling pathway,” and “neuroactive ligand-receptor interaction” were highly associated with the therapeutic pathway of Kangai injection (Table S4).

### 3.3. Potential Targets of Colorectal Cancer

A total of 7562 CRC-related genes were collected from GeneCards, Online Mendelian Inheritance in Man, and DisGeNET. All genes were ranked according to their scores, and only the top 300 genes were used for further analysis. The top 10 hub genes included “*TP53* (degree = 588),” “*AKT1* (degree = 525),” *EGFR* (degree = 491), “*MYC* (degree = 487),” “*GAPDH* (degree = 481),” “*VEGFA* (degree = 433),” “*INS* (degree = 417),” “*EGF* (degree = 403),” “*IL6* (degree = 400),” and “*PTEN* (degree = 392).” The Cytoscape software was used to construct the PPI network of CRC-related top 10 hub genes (Figure S1). The most significant GO-MF, GO-BP, GO-CC, and KEGG analysis were “enzyme binding,” “regulation of cell proliferation,” “nucleoplasm,” and “pathways in cancer,” respectively (Tables S5–S8).

### 3.4. Network Construction and Enrichment Analysis of Overlapping Genes

**Figure 2:**
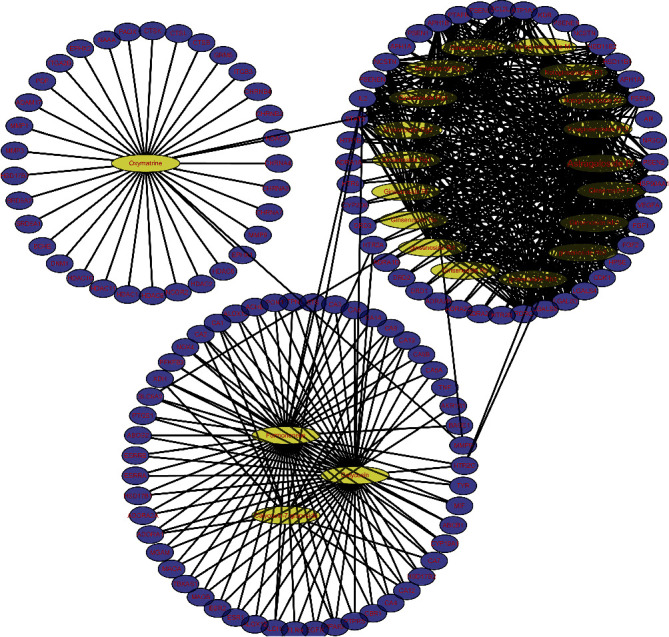
Composition of Kangai injection and its 155-gene target network.

**Figure 3:**
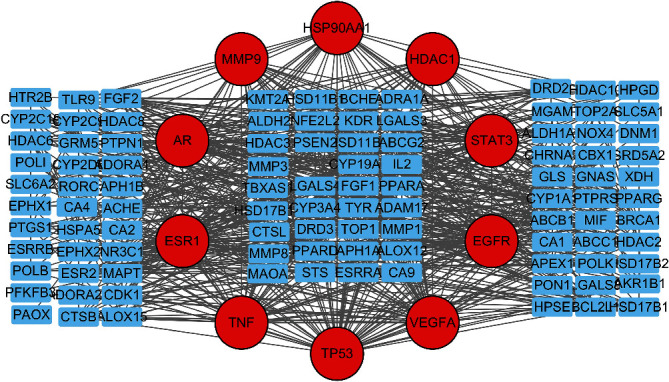
PPI network based on 114 overlapping genes.

Cytoscape was utilized to construct the network of active compound-potential targets of Kangai injection using 22 compounds and 150 targets, which contained 150 nodes and 575 edges (Figure 2). In order to visualize the mechanism of Kangai injection in the treatment of CRC, R software was used to determine the intersection of Kangai injection and CRC-related targets, and 114 overlapping genes were found. To further explore the pharmacological mechanism of Kangai injection in the treatment of CRC, we constructed a PPI network based on 114 overlapping genes (Figure 3); the red circles represented the top 10 hub genes which included “*TP53* (degree = 57),” “*VEGFA* (degree = 50),” “*EGFR* (degree = 49),” “*TNF* (degree = 43),” “*ESR1* (degree = 42),” “*STAT3* (degree = 39),” “*HSP90AA1* (degree = 36),” “*AR* (degree = 31),” “*HDAC1* (degree = 30),” and “*MMP9* (degree = 28).”

**Figure 4:**
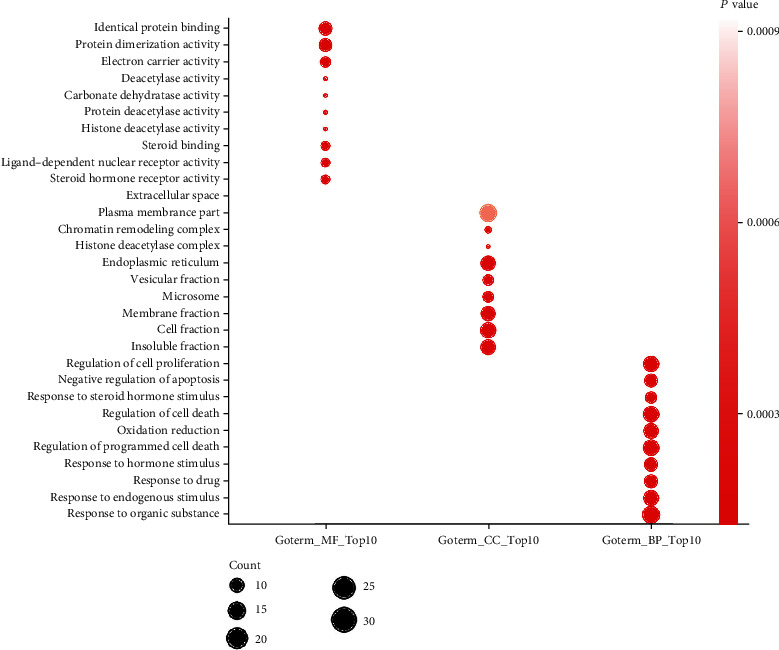
Top 10 GO entries of CC, MP, and BP.

**Figure 5:**
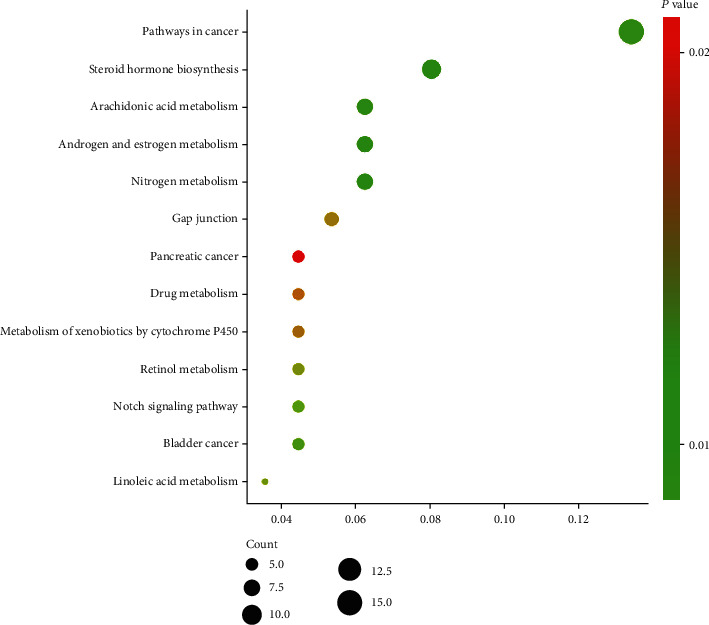
The Kyoto Encyclopedia of Genes and Genomes pathway.

**Figure 6:**
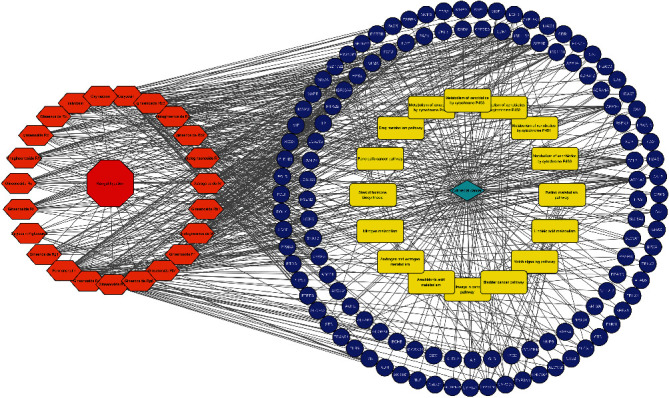
Construction of the drug-compound-target-pathway network.

A total of 244 Gene Ontology (GO) entries were obtained based on *P* value (<0.05), which included 19 of GO-cellular components (CC), 217 of GO-biological processes (BP), and 8 of GO-cellular components (CC). In GO-MF, potential targets were mainly concentrated in steroid hormone receptor activity, ligand-dependent nuclear receptor activity, steroid binding protein deacetylase activity, histone deacetylase activity, and so on (Figure 4). In GO-BP, potential targets were mainly enriched in response to organic substance, response to endogenous stimulus, response to drug, response to hormone stimulus, regulation of programmed cell death, and so on (Figure 4). In GO-CC, potential targets were mainly gathered in insoluble fraction, cell fraction, membrane fraction, microsome, vesicular fraction, and so on (Figure 4). 13 KEGG (the Kyoto Encyclopedia of Genes and Genomes pathway) entries in total were obtained based on *P* value (<0.05), which included steroid hormone biosynthesis, nitrogen metabolism, androgen and estrogen metabolism, arachidonic acid metabolism, pathways in cancer, bladder cancer, notch signaling pathway, linoleic acid metabolism, retinol metabolism, gap junction, metabolism of xenobiotics by cytochrome P450, drug metabolism, pancreatic cancer, and so on (Figure 5). Afterwards, we constructed a drug-compound-target-pathway interactive network including 22 compounds and 114 overlapping targets with 161 nodes and 707 edges (Figure 6).

### 3.5. Molecular Docking

**Figure 7:**
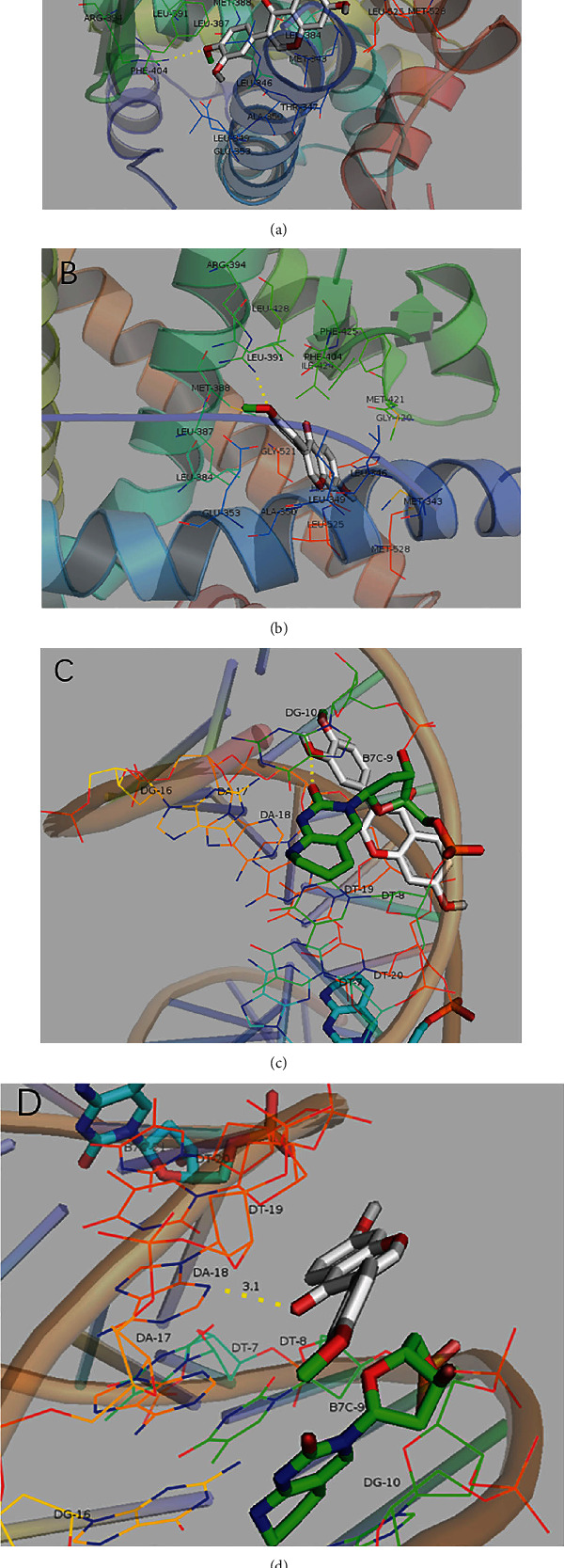
The top 4 of the molecular binding abilities (residues in red boxes are active site residues).

We searched the PubChem database for the 3D structures of all components of Kangai injection in the treatment of CRC. The top 10 hub genes in the PPI network and their corresponding components were docked (Table 2). As it was shown in Table 2, a total of 16 pairs of docking results were obtained. According to the binding force score, except that calycosin-7-glucoside and ginsenoside Rg4 had the weakest binding force with AR, the other 12 pairs of combinations all had strong ability to bind. The top 4 of the molecular bindings are shown in Figure 7.

## 4. Discussion

Kangai injection is a widely used TCM in clinical practice, and its components mainly include ginseng, astragali radix, and matrine [17]. In the present study, through a comprehensive and systematic literature searching, a total of 22 active ingredients of Kangai injection were collected, and 160 target proteins corresponding to these ingredients remained after deweighting; 7,562 target proteins related to CRC were obtained through the search; 114 target proteins were obtained from the intersection of target proteins of all ingredients of Kangai injection and CRC target proteins, which meant that these 114 target proteins might be the target proteins for CRC treatment by Kangai injection.

The top 10 hub target genes of Kangai injection were rated from high to low as *TP53*, *VEGFA*, *EGFR*, *TNF*, *ESR1*, *STAT3*, *HSP90AA1*, *HDAC1*, *AR*, and *MMP9*. Among the top 10 hub target genes of CRC and Kangai injection, there were 3 identical genes that were TP53, VEGFA, and EGFR in particular. Interestingly, the top 10 hub target genes of 114 overlapping genes between CRC and Kangai injection were identical with the top 10 hub target genes of Kangai injection. It is well known that as a tumor suppressor, TP53 plays an important role in the occurrence and development of almost all cancers, in CRC with no exception [34, 35]. The VEGF/VEGFR pathway mainly regulates vascular endothelial generation. VEGFA provides oxygen for tumor growth by promoting tumor angiogenesis [36–38]. The tumor microenvironment is closely related to the occurrence and development of cancer, while TNF is frequently present in the tumor microenvironment as an important inflammatory cytokine that promotes the invasion and metastasis of colon cancer [39]. STAT3 is able to promote epithelial mesenchymal transformation in CRC, thus promoting cancer metastasis [40].

In order to visually demonstrate the mechanism of Kangai injection in the treatment of CRC, we constructed an ingredient-target-disease interactive network including 22 compounds and 114 overlapping targets with 161 nodes and 707 edges. The network diagram also included key pathways for the treatment of CRC with Kangai injection. 13 KEGG entries in total were obtained based on the *P* value (<0.05). In the nitrogen metabolism pathway, overexpression of nitrogen permease regulator like-2 can increase the sensitivity of colon cancer cell lines to irinotecan [41]. The membrane androgen receptor (MAR) is expressed in colon cancer cells but rarely expressed in normal tissues. MAR can significantly regulate the invasion and metastasis of colon cancer cell lines [42]. Other signaling pathways also affect or regulate invasion and metastasis of colon cancer to a certain extent [43–45].

In this study, we observed that the components of Kangai injection could produce low molecular docking energy (-5.0 kcal/mol-7.8 kcal/mol) with CRC-related proteins *VEGFA*, *EGFR*, *TNF*, *ESR1*, *STAT3*, and so on, indicating that the components bind more tightly to these proteins. It was further confirmed that Kangai injection exerted anticolonial effects through a variety of cancer-related genes and pathways.

## 5. Conclusion

In the present study, Kangai injection plays a role in the treatment of CRC through multiple genes and pathways, providing a reliable basis for the clinical application of Kangai injection and for further research on the mechanism as well.

## Figures and Tables

**Table 1:** The components of Kangai injection.

Number	Compound	Molecular formula	Molecular weight	PubChem CID
1	Astragaloside IV	C_41_H_68_O_14_	785 g/mol	13943297
2	Calycosin	C_16_H_12_O_5_	284.26 g/mol	5280448
3	Calycosin-7-glucoside	C_22_H_22_O_10_	446.4 g/mol	71571502
4	Formononetin	C_16_H_12_O_4_	268.26 g/mol	5280378
5	Ginsenoside F1	C_36_H_62_O_9_	638.9 g/mol	9809542
6	Ginsenoside Rb1	C_54_H_92_O_23_	1109.3 g/mol	9898279
7	Ginsenoside Rb2	C_53_H_90_O_22_	1079.3 g/mol	6917976
8	Ginsenoside Rb3	C_53_H_90_O_22_	1079.3 g/mol	12912363
9	Ginsenoside Rc	C_53_H_90_O_22_	1079.3 g/mol	12855889
10	Ginsenoside Rd	C_48_H_82_O_19_	963.2 g/mol	24721561
11	Ginsenoside Re	C_48_H_82_O_18_	947.2 g/mol	441921
12	Ginsenoside Rf	C_42_H_72_O_14_	801 g/mol	441922
13	Ginsenoside Rg1	C_42_H_72_O_14_	801 g/mol	441923
14	Ginsenoside Rg2	C_42_H_72_O_13_	785 g/mol	21599924
15	Ginsenoside Rg4	C_42_H_70_O_12_	767 g/mol	102004835
16	Ginsenoside Rg6	C_42_H_70_O_12_	767 g/mol	91895489
17	Ginsenoside Rh1	C_36_H_62_O_9_	638.9 g/mol	12855920
18	Notoginsenoside M	C_48_H_82_O_19_	963.2 g/mol	85316219
19	Notoginsenoside R1	C_47_H_80_O_18_	933.1 g/mol	441934
20	Notoginsenoside R2	C_41_H_70_O_13_	771 g/mol	21599925
21	Oxymatrine	C_15_H_24_N_2_O_2_	264.4 g/mol	114850
22	Vinaginsenoside R13	C_48_H_84_O_20_	981.2 g/mol	73092886

**Table 2:** Molecular docking.

Hub genes	PDB enter ID	PDB ligand ID	Grid box dimension (center *x*, *y*, *z*)	Grid box size (*x*, *y*, *z*) (Å)	Compound	Binding energy (kcal/mol)
TP53	5ab9	92O	123.753, 104.322, -48.223	40, 40, 40 Å	Formononetin	-5.5
VEGFA	5 t89	NAG	-97.543, 23.676, 28.708	40, 40, 40 Å	Ginsenoside F1	-5.0
EGFR	6hve	GUW	-6.005, 14.401, 12.146	40, 40, 40 Å	Calycosin	-0.8
EGFR	6hve	GUW	-4.681, 5.36, 28.573	40, 40, 40 Å	Formononetin	-6.8
TNF	6ooz	A6Y	-12.439, -1.289, 18.733	40, 40, 40 Å	Calycosin-7-glucoside	-4.5
ESR1	1x7r	GEN	15.587, 32.224, 22.304	40, 40, 40 Å	Calycosin	-7.6
ESR1	1x7r	GEN	15.587, 32.224, 22.304	40, 40, 40 Å	Formononetin	-7.8
STAT3	6njs	KQV	13.498, 54.118, 0.1	40, 40, 40 Å	Ginsenoside F1	-5.5
HSP90AA1	2xjx	XJX	14.101, -2.869, 5.072	40, 40, 40 Å	Ginsenoside F1	-6.6
AR	3gjh	GAP	15.362, 4.093, 26.691	40, 40, 40 Å	Calycosin	-7.8
AR	3gjh	GAP	15.362, 4.093, 26.691	40, 40, 40 Å	Formononetin	-7.5
AR	2piq	RB1	-36.156, -9.368, 219.779	40, 40, 40 Å	Calycosin-7-glucoside	0
AR	4oed	DHT	-27.579, 2.536, -4.738	40, 40, 40 Å	Ginsenoside Rg4	26.4
HDAC1-	5w5k	K70	-16.926, 31.012, 3.329	40, 40, 40 Å	Oxymatrine	-7.0
MMP9	2ovx	4MR	71.85, 12.27, 54.262	40, 40, 40 Å	Ginsenoside Rg4	-7.2
MMP9	2ovz	5MR	70.536, 17.075, 53.277	40, 40, 40 Å	Oxymatrine	-6.9

## Data Availability

The data used to support the findings of this study are included within the article and supplementary information files.

## Supplementary Materials

Supplementary Materials Table S1: top 10-Gene Ontology-MF of potential therapeutic targets of Kangai injection. Table S2: top 10-Gene Ontology-CC of potential therapeutic targets of Kangai injection. Table S3: top 10-Gene Ontology-BP of potential therapeutic targets of Kangai injection. Table S4: KEGG of potential therapeutic targets of Kangai injection. Table S5: top 10-Gene Ontology-MF of top 300 target genes of colorectal cancer. Table S6: top 10-Gene Ontology-BP of top 300 target genes of colorectal cancer. Table S7: top 10-Gene Ontology-CC of top 300 target genes of colorectal cancer. Table S8: top 10-KEGG of top 300 target genes of colorectal cancer. Figure S1: the PPI network of colorectal cancer-related top 10 hub genes.
