# Supplementary material for: Network Pharmacology and Molecular Docking Validation to Reveal the Pharmacological Mechanisms of Kangai Injection against Colorectal Cancer
Source: Biomed Res Int. 2022 Aug 21;2022:3008842. doi: 10.1155/2022/3008842 (PMC9420643; doi:10.1155/2022/3008842)
Supplement: Supplementary Materials — Table S1: top 10-Gene Ontology-MF of potential therapeutic targets of Kangai injection. Table S2: top 10-Gene Ontology-CC of potential therapeutic targets of Kangai injection. Table S3: top 10-Gene Ontology-BP of potential therapeutic targets of Kangai injection. Table S4: KEGG of potential therapeutic targets of Kangai injection. Table S5: top 10-Gene Ontology-MF of top 300 target genes of colorectal cancer. Table S6: top 10-Gene Ontology-BP of top 300 target genes of colorectal cancer. Table S7: top 10-Gene Ontology-CC of top 300 target genes of colorectal cancer. Table S8: top 10-KEGG of top 300 target genes of colorectal cancer. Figure S1: the PPI network of colorectal cancer-related top 10 hub genes. [file 3008842.f1.docx]

S1 Table: TOP10-Gene ontology-MF of potential therapeutic targets of kangai injection

| Term | Count | % | P Value | Genes | FDR |
| --- | --- | --- | --- | --- | --- |
| Carbonate dehydratase activity | 12 | 0.665188 | 1.30E-18 | CA9, CA14, CA13, CA7, CA5A, CA12, CA6, CA5B, CA4, CA3, CA2, CA1 | 1.84E-15 |
| Amine receptor activity | 13 | 0.720621 | 1.98E-14 | HTR1B, DRD1, DRD3, DRD2, HTR6, ADRA2A, ADRA1A, ADRA2C, ADRA2B, HTR2B, HTR2C, ADRA1D, HTR2A | 2.80E-11 |
| Hydro-lyase activity | 13 | 0.720621 | 5.13E-14 | CA14, CA13, CA5A, CA12, CA5B, CA9, CA7, CA6, CA4, ABCC1, CA3, CA2, CA1 | 7.28E-11 |
| Amine binding | 14 | 0.776053 | 3.61E-10 | ACHE, DRD1, DRD3, DRD2, HTR1B, BCHE, CHRNB4, CHRNA4, CHRNA7, CHRNB2, HTR2B, HTR2C, CHRNA3, HTR2A | 5.12E-07 |
| Steroid hormone receptor activity | 10 | 0.554324 | 3.77E-09 | PPARA, PPARD, AR, ESRRA, ESRRB, PPARG, ESR1, RORC, NR3C1, ESR2 | 5.34E-06 |
| Histone deacetylase activity | 7 | 0.388027 | 9.49E-09 | HDAC3, HDAC2, HDAC1, HDAC10, HDAC11, HDAC8, HDAC6 | 1.35E-05 |
| Protein deacetylase activity | 7 | 0.388027 | 9.49E-09 | HDAC3, HDAC2, HDAC1, HDAC10, HDAC11, HDAC8, HDAC6 | 1.35E-05 |
| Ligand-dependent nuclear receptor activity | 10 | 0.554324 | 1.79E-08 | PPARA, PPARD, AR, ESRRA, ESRRB, PPARG, ESR1, RORC, NR3C1, ESR2 | 2.54E-05 |
| Deacetylase activity | 7 | 0.388027 | 3.42E-08 | HDAC3, HDAC2, HDAC1, HDAC10, HDAC11, HDAC8, HDAC6 | 4.86E-05 |
| Steroid binding | 10 | 0.554324 | 4.36E-08 | CYP3A4, ALDH1A1, HSD17B10, AR, ESRRA, ESRRB, ESR1, HSD11B2, NR3C1, ESR2 | 6.19E-05 |

MF: molecular function, FDR: False discovery rate

S2 Table: TOP10-Gene ontology-CC of potential therapeutic targets of kangai injection

| Term | Count | % | P Value | Genes | FDR |
| --- | --- | --- | --- | --- | --- |
| Plasma membrane part | 57 | 3.159645 | 6.96E-11 | SLC5A2, RAB9A, SLC6A2, SLC5A1, ADORA1, HTR1B, MAPT, CHRNA4, CHRNA7, PSENEN, CHRNA3, EGFR, ESR1, PTPRS, GRM5, NPC1, PSEN1, CA9, BACE1, HTR6, PSEN2, ADAM17, CA4, GNAS, CTSB, CA2, PTAFR, ITGA2B, DRD1, ACHE, TNF, APH1A, DRD3, ADORA2A, DRD2, ITGB3, BCL2L1, ADRA2A, ADRA2C, ADRA2B, NOX4, MAOA, MAOB, ABCB1, ATP1A1, KDR, NCSTN, CHRNB4, ADRA1A, ABCC1, CHRNB2, PTPN1, HTR2B, HTR2C, ADRA1D, HDAC6, HTR2A | 9.10E-08 |
| Integral to plasma membrane | 38 | 2.10643 | 1.95E-09 | DRD1, TNF, APH1A, DRD3, SLC6A2, ADORA2A, DRD2, SLC5A1, ITGB3, ADORA1, HTR1B, ADRA2A, CHRNA4, CHRNA7, PSENEN, ADRA2C, ADRA2B, CHRNA3, PTPRS, ATP1A1, KDR, NCSTN, GRM5, NPC1, PSEN1, BACE1, HTR6, PSEN2, CHRNB4, ADRA1A, ADAM17, ABCC1, CHRNB2, HTR2B, ADRA1D, PTAFR, HTR2A, ITGA2B | 2.56E-06 |
| Intrinsic to plasma membrane | 38 | 2.10643 | 3.62E-09 | DRD1, TNF, APH1A, DRD3, SLC6A2, ADORA2A, DRD2, SLC5A1, ITGB3, ADORA1, HTR1B, ADRA2A, CHRNA4, CHRNA7, PSENEN, ADRA2C, ADRA2B, CHRNA3, PTPRS, ATP1A1, KDR, NCSTN, GRM5, NPC1, PSEN1, BACE1, HTR6, PSEN2, CHRNB4, ADRA1A, ADAM17, ABCC1, CHRNB2, HTR2B, ADRA1D, PTAFR, HTR2A, ITGA2B | 4.74E-06 |
| Insoluble fraction | 31 | 1.718404 | 4.22E-09 | CYP3A4, DRD1, CYP2C19, DRD3, SLC6A2, ADORA2A, CYP2D6, PTGS1, BCHE, CHRNA4, SRD5A1, SRD5A2, CYP19A1, STS, TBXAS1, CYP1A1, CYP2C9, TP53, EPHX1, ABCB1, ATP1A1, NCSTN, PSEN1, PSEN2, BACE1, PON1, CA4, HSD11B2, GNAS, ABCC1, PTPN1 | 5.51E-06 |
| Endoplasmic reticulum | 33 | 1.829268 | 6.36E-09 | CYP3A4, HSD17B10, DRD1, RAB9A, APH1A, HSD17B2, CYP2C19, CYP2D6, PTGS1, ADORA1, BCHE, PSENEN, SRD5A1, HSPA5, SRD5A2, APEX1, HSD17B7, CYP19A1, NOX4, STS, CYP1A1, CYP2C9, TP53, EPHX1, NCSTN, NPC1, PSEN1, BACE1, PSEN2, HSD11B1, CA4, HSD11B2, PTPN1 | 8.32E-06 |
| Membrane fraction | 29 | 1.607539 | 3.10E-08 | CYP3A4, DRD1, CYP2C19, DRD3, SLC6A2, ADORA2A, CYP2D6, PTGS1, BCHE, CHRNA4, SRD5A1, SRD5A2, CYP19A1, STS, TBXAS1, CYP1A1, CYP2C9, EPHX1, ABCB1, ATP1A1, NCSTN, PSEN1, PSEN2, BACE1, PON1, CA4, HSD11B2, GNAS, ABCC1 | 4.05E-05 |
| Cell fraction | 33 | 1.829268 | 1.10E-07 | CYP3A4, DRD1, CYP2C19, DRD3, SLC6A2, ADORA2A, CYP2D6, PTGS1, BCHE, CHRNA4, SRD5A1, SRD5A2, CYP19A1, STS, TBXAS1, CYP1A1, CYP2C9, EPHX2, TP53, EPHX1, ABCB1, ATP1A1, NCSTN, PSEN1, BACE1, PSEN2, PON1, CA4, HSD11B2, GNAS, ABCC1, CTSB, PTPN1 | 1.44E-04 |
| Plasma membrane | 71 | 3.935698 | 1.29E-07 | SLC5A2, RAB9A, SLC6A2, SLC5A1, PTGS1, ADORA1, TLR9, HTR1B, MAPT, CHRNA4, CHRNA7, PSENEN, CHRNA3, EGFR, STS, CA12, ESR1, PTPRS, HDAC11, GRM5, NPC1, PSEN1, CA9, BACE1, PSEN2, MGAM, HTR6, ADAM17, CA4, GNAS, CTSB, CA2, PTAFR, ALOX12, ITGA2B, HSD17B10, ACHE, DRD1, TNF, APH1A, DRD3, ADORA2A, DRD2, APH1B, ITGB3, BCL2L1, ADRA2A, ADRA2C, ADRA2B, HSD17B7, NOX4, LGALS3, LGALS4, MAOA, MAOB, ABCB1, ATP1A1, STAT3, KDR, ABCG2, NCSTN, CHRNB4, ADRA1A, ABCC1, CHRNB2, PTPN1, HTR2B, HTR2C, ADRA1D, HDAC6, HTR2A | 1.68E-04 |
| Microsome | 14 | 0.776053 | 1.67E-06 | CYP3A4, STS, TBXAS1, CYP1A1, CYP2C19, CYP2C9, PTGS1, CYP2D6, EPHX1, ATP1A1, PON1, HSD11B2, SRD5A1, SRD5A2 | 0.002188 |
| Vesicular fraction | 14 | 0.776053 | 2.31E-06 | CYP3A4, STS, TBXAS1, CYP1A1, CYP2C19, CYP2C9, PTGS1, CYP2D6, EPHX1, ATP1A1, PON1, HSD11B2, SRD5A1, SRD5A2 | 0.003026 |

CC: cell component, FDR: False discovery rate

S3 Table: TOP10-Gene ontology-BP of potential therapeutic targets of kangai injection

| Term | Count | % | P Value | Genes | FDR |
| --- | --- | --- | --- | --- | --- |
| Response to organic substance | 42 | 2.32816 | 4.99E-19 | PPARA, DRD1, TNF, DRD3, ADORA2A, DRD2, PPARG, PTGS1, BCL2L1, NR3C1, MMP3, HTR1B, BCHE, CHRNA4, CHRNA7, SRD5A2, CHRNA3, EGFR, STS, AR, HSP90AA1, CYP1A1, ESR1, EPHX1, ESR2, BRCA1, STAT3, HDAC2, CA9, ALDH2, CHRNB4, HSD11B2, ADAM17, CA4, CHRNB2, GNAS, PTPN1, NFE2L2, CA2, HTR2C, PTAFR, HDAC6 | 8.60E-16 |
| Response to drug | 25 | 1.385809 | 9.38E-18 | DRD1, DRD3, DRD2, PPARG, TOP1, BCHE, SRD5A1, SRD5A2, CHRNA3, CYP1A1, TP53, ABCB1, ATP1A1, STAT3, ABCG2, HDAC3, HDAC2, CA9, ADAM17, HSD11B2, CA4, ABCC1, GNAS, HTR2C, HTR2A | 1.62E-14 |
| Response to alkaloid | 15 | 0.831486 | 4.09E-16 | DRD1, DRD3, ADORA2A, DRD2, PPARG, HTR1B, HDAC2, BCHE, ALDH2, CHRNB4, CHRNA4, CHRNA7, CHRNB2, HTR2C, CHRNA3 | 7.66E-13 |
| Response to organic cyclic substance | 19 | 1.053215 | 8.16E-16 | STS, DRD1, CYP1A1, DRD3, ADORA2A, DRD2, PPARG, EPHX1, BCL2L1, STAT3, HTR1B, HDAC2, CHRNB4, ALDH2, CHRNA4, CHRNA7, CHRNB2, HTR2C, CHRNA3 | 1.34E-12 |
| Regulation of amine transport | 12 | 0.665188 | 2.91E-14 | HTR1B, DRD1, TNF, DRD3, ADORA2A, DRD2, PTGS1, CHRNA4, CHRNB2, HTR2C, CHRNA3, HTR2A | 5.01E-11 |
| Response to endogenous stimulus | 28 | 1.552106 | 3.20E-14 | PPARA, DRD1, TNF, DRD3, ADORA2A, DRD2, PPARG, PTGS1, BCL2L1, MMP3, HTR1B, BCHE, SRD5A2, AR, STS, ESR1, ESR2, BRCA1, STAT3, CA9, ALDH2, ADAM17, CA4, HSD11B2, GNAS, CA2, PTPN1, HDAC6 | 5.53E-11 |
| Regulation of synaptic transmission | 17 | 0.94235 | 1.58E-12 | EGFR, ACHE, DRD1, TNF, DRD3, ADORA2A, DRD2, ADORA1, HTR1B, PSEN1, BCHE, PSEN2, CHRNB4, CHRNB2, HTR2C, CHRNA3, HTR2A | 2.72E-09 |
| Regulation of transmission of nerve impulse | 17 | 0.94235 | 5.32E-12 | EGFR, ACHE, DRD1, TNF, DRD3, ADORA2A, DRD2, ADORA1, HTR1B, PSEN1, BCHE, PSEN2, CHRNB4, CHRNB2, HTR2C, CHRNA3, HTR2A | 9.16E-09 |
| Regulation of neurological system process | 17 | 0.94235 | 9.86E-12 | EGFR, ACHE, DRD1, TNF, DRD3, ADORA2A, DRD2, ADORA1, HTR1B, PSEN1, BCHE, PSEN2, CHRNB4, CHRNB2, HTR2C, CHRNA3, HTR2A | 1.70E-08 |
| Positive regulation of catalytic activity | 28 | 1.552106 | 1.20E-11 | DRD1, TNF, DRD3, APH1A, ADORA2A, DRD2, APH1B, ADORA1, ADRA2A, CHRNA7, PSENEN, ADRA2C, ADRA2B, FGF2, CHRNA3, EGFR, CDK1, TP53, NCSTN, GRM5, PSEN1, PSEN2, ADAM17, GNAS, HTR2B, HTR2C, HTR2A, IL2 | 2.06E-08 |

BP: biological process, FDR: False discovery rate

S4 Table: KEGG of potential therapeutic targets of kangai injection

| Term | Count | % | P Value | Genes | FDR |
| --- | --- | --- | --- | --- | --- |
| Nitrogen metabolism | 13 | 0.720621 | 7.08E-15 | CA14, CA13, CA5A, CA12, CA5B, CA9, CA7, GLS, CA6, CA4, CA3, CA2, CA1 | 8.16E-12 |
| Steroid hormone biosynthesis | 11 | 0.609756 | 3.45E-08 | CYP3A4, STS, HSD17B2, CYP1A1, HSD17B1, HSD11B1, HSD11B2, SRD5A1, SRD5A2, HSD17B7, CYP19A1 | 3.96E-05 |
| Androgen and estrogen metabolism | 9 | 0.498891 | 9.12E-07 | STS, HSD17B2, HSD17B1, HSD11B1, HSD11B2, SRD5A1, SRD5A2, HSD17B7, CYP19A1 | 0.001047 |
| Notch signaling pathway | 9 | 0.498891 | 6.20E-06 | NCSTN, HDAC2, PSEN1, HDAC1, APH1A, PSEN2, ADAM17, PSENEN, NCOR2 | 0.007115 |
| Neuroactive ligand-receptor interaction | 18 | 0.997783 | 3.21E-05 | DRD1, DRD3, ADORA2A, DRD2, NR3C1, ADORA1, GRM5, HTR1B, HTR6, ADRA2A, ADRA1A, ADRA2C, ADRA2B, HTR2B, HTR2C, PTAFR, ADRA1D, HTR2A | 0.036828 |

KEGG: Kyoto Encyclopedia of Genes and Genomes, FDR: False discovery rate

S5 Table: TOP10-Gene ontology-MF of TOP300 target genes of colorectal cancer

| Term | Count | % | P Value | Genes | FDR |
| --- | --- | --- | --- | --- | --- |
| Enzyme binding | 58 | 1.33733 | 1.11E-24 | AURKA, TLR4, FOXO3, CTNNB1, WNT2, AKT1, CDKN2A, HMOX1, SERPINE1, H2AFX, PRKACA, FAS, TOP2A, MAP2K7, EGFR, RELA, SOCS1, TP53, RB1, CD40, IRS1, PRKCD, MAPK1, CCND1, HIF1A, CCND2, LCK, MDM2, CSF3, PRKCZ, CAV1, ERBB2, NFKBIA, CHEK1, CDH1, CDH2, TCF7L2, IGF1R, RAC1, CD24, AXIN2, PIK3R1, ACTB, MAP2K1, FLT3, SMAD3, SMAD2, FOXP3, STAT3, BRCA1, PTPN11, VWF, HDAC2, HDAC1, SP1, GSK3B, JAK2, SELE | 1.64E-21 |
| Protein kinase activity | 60 | 1.383445 | 5.09E-23 | RPS6KB1, AURKA, WNT2, AKT1, PRKACA, MAP2K7, AKT3, AKT2, PRKCA, EGFR, CDK1, RET, CDK6, ALK, CDK4, IRS1, PRKCD, CDK2, MAPK1, CCND1, MAPK3, LCK, PDGFRA, PDGFRB, MAPK9, MAPK8, FGFR2, PRKCZ, FGFR1, CCL2, FGFR3, ERBB4, ERBB3, STK11, ERBB2, CHEK1, KIT, CHEK2, SRC, IGF1R, PTK2, TEK, CSF1R, FLT1, MAP2K1, FLT3, MET, RAF1, ATR, MAPK10, ATM, KDR, PLK1, MAPK14, GSK3B, JAK1, JAK2, MTOR, ABL1, ENG | 7.52E-20 |
| Kinase binding | 32 | 0.737837 | 1.66E-19 | PRKCZ, CAV1, CHEK1, TLR4, FOXO3, CDH2, TCF7L2, CTNNB1, WNT2, IGF1R, CDKN2A, PRKACA, FAS, CD24, AXIN2, MAP2K7, TOP2A, ACTB, MAP2K1, FLT3, RELA, SOCS1, SMAD3, RB1, IRS1, STAT3, MAPK1, CCND1, CCND2, GSK3B, LCK, JAK2 | 2.45E-16 |
| Identical protein binding | 56 | 1.291215 | 6.21E-19 | PDGFB, NFKB1, TGFB1, AKT1, ACTG1, APOE, CASP8, CAT, FAS, TOP2A, TERT, EGFR, HSP90AA1, RELA, CDK2, RAD51, HNF4A, VEGFA, PDGFRA, HSPB1, MDM2, SMARCA4, CAV1, TNF, FGFR3, ERBB3, ERBB2, CSF1, KITLG, NFKBIA, ITGB3, BCL2L1, ITGB1, IGF1R, NPM1, THBS1, AXIN1, CSF1R, BMP4, BMP2, CEBPB, FLT1, MSH2, SMAD4, SMAD3, FOXP3, SIRT1, ADIPOQ, BRCA1, ABCG2, VWF, HDAC1, SP1, TSC2, PARP1, ENG | 9.16E-16 |
| Growth factor activity | 29 | 0.668665 | 9.08E-18 | CXCL1, CSF3, CSF2, FGF8, PDGFB, CSF1, KITLG, JAG1, CXCL12, TGFB1, IL10, BDNF, INS, IL1B, EGF, FGF2, IL4, BMP4, IL6, BMP2, IL7, IGF1, IGF2, HGF, LEP, VEGFC, DKK1, VEGFA, NGF, IL2 | 1.34E-14 |
| Protein tyrosine kinase activity | 29 | 0.668665 | 2.09E-17 | FGFR2, FGFR1, FGFR3, ERBB4, ERBB3, ERBB2, KIT, SRC, WNT2, IGF1R, PTK2, TEK, MAP2K7, CSF1R, EGFR, RET, FLT1, MAP2K1, FLT3, MET, ALK, IRS1, KDR, LCK, PDGFRA, PDGFRB, JAK1, JAK2, ABL1 | 3.09E-14 |
| Structure-specific DNA binding | 27 | 0.62255 | 6.44E-17 | XRCC5, CDX2, XRCC6, STAT5B, MLH1, ZEB1, WT1, CTNNB1, FOS, MYC, ERCC1, EGR1, EGFR, EXO1, MSH6, MSH2, CREB1, TP53, SMAD3, BRCA2, SMAD2, RAD51, NOTCH1, SP1, JUN, PCNA, KLF4 | 1.67E-13 |
| Double-stranded DNA binding | 23 | 0.53032 | 9.17E-17 | EGFR, EGR1, XRCC5, MSH6, CDX2, MSH2, CREB1, XRCC6, STAT5B, TP53, MLH1, SMAD3, SMAD2, ZEB1, WT1, CTNNB1, RAD51, FOS, SP1, JUN, PCNA, MYC, KLF4 | 1.67E-13 |
| Transmembrane receptor protein tyrosine kinase activity | 20 | 0.461148 | 1.32E-16 | EGFR, FGFR2, FGFR1, RET, FGFR3, FLT1, ERBB4, ERBB3, FLT3, ERBB2, MET, KIT, ALK, IRS1, KDR, IGF1R, TEK, PDGFRA, PDGFRB, CSF1R | 1.67E-13 |
| Transcription activator activity | 40 | 0.922297 | 6.09E-15 | E2F1, XRCC6, PPARG, FOXO1, CDH1, FOXO3, ZEB1, SOX9, WT1, TGFB1, CTNNB1, GLI1, CCNE1, NPM1, YAP1, RUNX2, EGR1, AR, CEBPB, CREBBP, SMAD4, SMAD3, LEF1, BRCA2, SMAD2, RB1, KAT5, FOXP3, BRCA1, STAT3, CD86, NOTCH1, EP300, HDAC2, SP1, HNF4A, HDAC1, CD80, KLF4, SMARCA4 | 9.02E-12 |

MF: molecular function, FDR: False discovery rate

S6 Table: TOP10-Gene ontology-BP of TOP300 target genes of colorectal cancer

| Term | Count | % | P Value | Genes | FDR |
| --- | --- | --- | --- | --- | --- |
| Regulation of cell proliferation | 134 | 3.089693 | 2.50E-86 | HRAS, CDX2, PDGFB, PTGS2, STAT5B, MMP7, IL13, FOXO1, RPS6KB1, JAG1, IL15, IL10, TGFB1, SHH, CTNNB1, WNT2, PGR, BDNF, CDKN2A, APOE, IFNG, SERPINE1, IL1B, CCNA2, EGFR, RELA, CD40, IRS1, MAPK1, VEGFC, HIF1A, HNF4A, JUN, VEGFA, PDGFRA, PDGFRB, NGF, FGFR2, FGFR1, CAV1, CCL2, FGFR3, ERBB4, STK11, ERBB3, ERBB2, SOX2, NFKBIA, CHEK1, BCL2L1, SOX9, ITGB1, TIMP1, KRAS, INS, TEK, NPM1, EGF, AXIN2, RUNX2, RUNX3, BMP4, BMP2, BECN1, SMAD4, BRCA2, SMAD3, SMAD2, FOXP3, BRCA1, KDR, CDKN1A, NOTCH1, CDKN1B, HDAC2, HDAC1, ETS1, TSC2, CD274, MTOR, ENG, KLF4, BMI1, PPARG, ZEB1, PTEN, GLI1, CASP3, HMOX1, NOS3, SHC1, FGF2, MYC, PRKCA, TP53, CDK6, RB1, CDK4, CDK2, MYCN, CD86, CCND1, CD80, CCND2, MDM2, CXCL1, CSF3, CSF2, PRKCZ, TNF, CSF1, FOXM1, KITLG, KIT, STAT6, IGF1R, CD24, THBS1, IL4, IL6, FLT1, IL7, FLT3, NF1, CTLA4, IGF1, IGF2, STAT1, CDKN3, SIRT1, PLG, SOD2, NRAS, JAK2, IL2 | 4.55E-83 |
| Positive regulation of macromolecule metabolic process | 121 | 2.789947 | 5.67E-67 | HRAS, CDX2, PDGFB, STAT5B, TLR1, XRCC6, TLR3, FOXO1, TLR4, FOXO3, TLR7, IL10, TGFB1, TLR8, SHH, TLR9, CTNNB1, WNT1, APOE, IFNG, IL1B, H2AFX, CCNA2, RELA, CD40, IRS1, MAPK1, VEGFC, EP300, HIF1A, HNF4A, JUN, VEGFA, F2, PDGFRA, MAPK9, SMARCA4, CAV1, SOX2, NFKBIA, SOX9, WT1, IL17A, SMARCB1, INS, RUNX2, AXIN1, BMP4, BMP2, MAP2K1, CREBBP, SMAD4, SMAD3, SMAD2, FOXP3, KAT5, BRCA1, NOTCH1, HDAC2, HDAC1, PLK1, ETS1, DNMT1, MTOR, ENG, KLF4, E2F1, PPARG, NFKB1, ZEB1, GLI1, AKT1, CCNE1, FOS, HMOX1, SHC1, YAP1, FGF2, MYC, AKT2, EGR1, PRKCA, CDK1, AR, ESR1, TP53, LEF1, RB1, CDK4, CDK2, RAD51, CD86, CCND1, CD80, CCND2, MDM2, CSF2, TNF, CSF1, KITLG, KIT, TCF7L2, STAT6, TNFRSF1A, IGF1R, CD24, THBS1, IL4, IL6, CEBPB, CREB1, IGF1, IGF2, ADIPOQ, STAT3, CCNB1, SP1, MAPK14, IRF1, JAK2, SELE, IL2 | 1.03E-63 |
| Regulation of cell death | 118 | 2.720775 | 2.65E-66 | XRCC5, HRAS, PTGS2, MMP9, STAT5B, TLR2, FOXO1, TLR4, FOXO3, IL10, TGFB1, SHH, BDNF, CDKN2A, CD44, APOE, IFNG, IL1B, PIK3CA, FAS, EGFR, RELA, BCL2L11, MAPK1, CD40LG, JUN, VEGFA, F2, MAPK9, HSPB1, MAPK8, NGF, HMGB1, CCL2, MCL1, ERBB3, ERBB2, NFKBIA, BCL2L1, CHEK2, SOX9, SRC, KRAS, ALB, INS, RAC1, NPM1, RUNX3, BMP4, BECN1, BRCA2, SMAD3, HGF, BRCA1, ATM, CDKN1A, NOTCH1, CDKN1B, HDAC1, ETS1, ABL1, MLH1, FASLG, NFKB1, PTEN, AKT1, CASP3, CASP9, HMOX1, CASP8, RHOA, NOS3, CAT, FGF2, MYC, TOP2A, TERT, PRKCA, PPP2R1A, CDK1, CYCS, ESR1, TP53, LCK, CSF2, PRKCZ, TNF, XIAP, MGMT, CDH1, KIT, TCF7L2, IGF1R, SOS1, CD24, HSPA5, THBS1, ERCC1, IL4, MSH6, IL6, CEBPB, IL7, MSH2, CREB1, NF1, IGF1, IGF2, ANXA5, STAT1, ADIPOQ, SIRT1, PLG, SOD2, NRAS, GSK3B, MPO, JAK2, IL2 | 4.83E-63 |
| Regulation of programmed cell death | 117 | 2.697717 | 2.03E-65 | XRCC5, HRAS, PTGS2, MMP9, STAT5B, TLR2, FOXO1, TLR4, FOXO3, IL10, TGFB1, SHH, BDNF, CDKN2A, CD44, APOE, IFNG, PIK3CA, IL1B, FAS, EGFR, RELA, BCL2L11, MAPK1, CD40LG, JUN, VEGFA, F2, MAPK9, HSPB1, MAPK8, NGF, HMGB1, CCL2, MCL1, ERBB3, ERBB2, NFKBIA, BCL2L1, CHEK2, SOX9, SRC, KRAS, ALB, INS, RAC1, NPM1, RUNX3, BECN1, BRCA2, SMAD3, HGF, BRCA1, ATM, CDKN1A, NOTCH1, CDKN1B, HDAC1, ETS1, ABL1, MLH1, FASLG, NFKB1, PTEN, AKT1, CASP3, CASP9, HMOX1, CASP8, RHOA, NOS3, CAT, FGF2, MYC, TOP2A, TERT, PRKCA, PPP2R1A, CDK1, CYCS, ESR1, TP53, LCK, CSF2, PRKCZ, TNF, XIAP, MGMT, CDH1, KIT, TCF7L2, IGF1R, SOS1, CD24, HSPA5, THBS1, ERCC1, IL4, MSH6, IL6, CEBPB, IL7, MSH2, CREB1, NF1, IGF1, IGF2, ANXA5, STAT1, ADIPOQ, SIRT1, PLG, SOD2, NRAS, GSK3B, MPO, JAK2, IL2 | 3.70E-62 |
| Regulation of apoptosis | 115 | 2.651602 | 8.85E-64 | XRCC5, HRAS, PTGS2, MMP9, STAT5B, TLR2, FOXO1, TLR4, FOXO3, IL10, TGFB1, SHH, BDNF, CDKN2A, CD44, APOE, IFNG, PIK3CA, IL1B, FAS, EGFR, RELA, BCL2L11, MAPK1, CD40LG, JUN, VEGFA, F2, MAPK9, HSPB1, MAPK8, NGF, HMGB1, CCL2, MCL1, ERBB3, ERBB2, NFKBIA, BCL2L1, CHEK2, SOX9, SRC, KRAS, ALB, INS, RAC1, NPM1, RUNX3, BECN1, BRCA2, SMAD3, HGF, BRCA1, ATM, NOTCH1, CDKN1A, CDKN1B, HDAC1, ETS1, ABL1, MLH1, FASLG, NFKB1, PTEN, AKT1, CASP3, CASP9, HMOX1, CASP8, RHOA, NOS3, CAT, MYC, TOP2A, TERT, PRKCA, PPP2R1A, CDK1, CYCS, ESR1, TP53, LCK, CSF2, PRKCZ, TNF, XIAP, MGMT, CDH1, TCF7L2, IGF1R, SOS1, CD24, HSPA5, THBS1, ERCC1, IL4, MSH6, IL6, CEBPB, IL7, MSH2, CREB1, NF1, IGF1, IGF2, ANXA5, STAT1, ADIPOQ, SIRT1, PLG, SOD2, NRAS, GSK3B, MPO, JAK2, IL2 | 1.61E-60 |
| Response to organic substance | 106 | 2.444086 | 2.39E-59 | PTGS2, PDGFB, STAT5B, TLR2, TLR3, FOXO1, RPS6KB1, AURKA, TLR4, MMP3, IL10, TGFB1, SHH, CTNNB1, WNT2, CD44, APOE, GATA3, IFNG, IL1B, PRKACA, FAS, CCNA2, EGFR, RELA, SOCS1, IRS1, MAPK1, EP300, HNF4A, JUN, PDGFRA, HSPB1, NGF, CAV1, CCL2, ERBB4, MCL1, GRB2, ERBB3, ERBB2, NFKBIA, BCL2L1, SRC, KRAS, INS, IDH1, SPP1, BMP4, MAP2K1, BRCA2, SMAD3, SMAD2, BRCA1, PTPN11, CDKN1A, HDAC2, MTOR, PARP1, DICER1, PPARG, FASLG, PTEN, AKT1, CCNE1, FOS, CASP3, HMOX1, CASP8, NOS3, SHC1, MYC, AKT2, PRKCA, EGR1, PPP2R1A, AR, HSP90AA1, ESR1, LEF1, MMP14, CCND1, CCND2, TNF, CDH1, STAT6, TNFRSF1A, IGF1R, HSPA4, CD24, THBS1, HSPA8, PIK3R1, IL6, MSH2, SELL, CREB1, IGF2, ANXA5, STAT1, ADIPOQ, STAT3, LEP, AFP, MAPK14, JAK2, SELE | 4.36E-56 |
| Positive regulation of biosynthetic process | 104 | 2.397971 | 7.34E-59 | HRAS, CDX2, PDGFB, STAT5B, TLR1, XRCC6, TLR3, FOXO1, TLR4, FOXO3, TLR7, TLR8, IL10, TGFB1, SHH, TLR9, CTNNB1, WNT1, APOE, IFNG, IL1B, CCNA2, EGFR, RELA, IRS1, MAPK1, HIF1A, EP300, HNF4A, JUN, VEGFA, F2, PDGFRA, SMARCA4, SOX2, NFKBIA, SOX9, WT1, IL17A, SMARCB1, INS, NPM1, RUNX2, BMP4, BMP2, CREBBP, SMAD4, SMAD3, SMAD2, FOXP3, KAT5, BRCA1, NOTCH1, HDAC2, HDAC1, ETS1, MTOR, ENG, KLF4, E2F1, PPARG, NFKB1, ZEB1, GLI1, AKT1, CCNE1, FOS, HMOX1, SHC1, YAP1, FGF2, MYC, AKT2, EGR1, ICAM1, AR, HSP90AA1, TP53, LEF1, RB1, CDK4, CDK2, CD86, CD80, CSF2, TNF, KITLG, TCF7L2, STAT6, TNFRSF1A, IGF1R, THBS1, IL4, IL6, CEBPB, CREB1, IGF1, IGF2, STAT3, SOD2, SP1, MAPK14, IRF1, JAK2, IL2 | 1.34E-55 |
| Positive regulation of cellular biosynthetic process | 103 | 2.374914 | 1.96E-58 | E2F1, HRAS, CDX2, PDGFB, XRCC6, TLR1, STAT5B, PPARG, FOXO1, TLR3, TLR4, NFKB1, ZEB1, FOXO3, TLR7, SHH, TGFB1, IL10, TLR8, GLI1, CTNNB1, TLR9, AKT1, WNT1, FOS, CCNE1, APOE, HMOX1, IFNG, IL1B, SHC1, YAP1, CCNA2, MYC, FGF2, AKT2, EGR1, EGFR, ICAM1, AR, HSP90AA1, RELA, TP53, LEF1, RB1, CDK4, IRS1, CDK2, MAPK1, CD86, EP300, HIF1A, HNF4A, CD80, JUN, VEGFA, PDGFRA, SMARCA4, CSF2, TNF, SOX2, KITLG, NFKBIA, SOX9, TCF7L2, WT1, STAT6, IGF1R, TNFRSF1A, IL17A, INS, SMARCB1, NPM1, THBS1, RUNX2, BMP4, IL4, IL6, BMP2, CEBPB, CREB1, CREBBP, SMAD4, IGF1, SMAD3, IGF2, SMAD2, KAT5, FOXP3, STAT3, BRCA1, SOD2, NOTCH1, HDAC2, SP1, HDAC1, ETS1, MAPK14, IRF1, JAK2, MTOR, ENG, KLF4, IL2 | 3.56E-55 |
| Positive regulation of cell proliferation | 82 | 1.890708 | 4.02E-55 | BMI1, HRAS, CDX2, PDGFB, PTGS2, STAT5B, IL13, RPS6KB1, IL15, SHH, TGFB1, GLI1, IFNG, IL1B, SHC1, FGF2, MYC, CCNA2, PRKCA, EGFR, RELA, CDK6, CD40, CDK4, IRS1, CDK2, MYCN, MAPK1, VEGFC, CD86, CCND1, HIF1A, CD80, CCND2, JUN, VEGFA, PDGFRA, PDGFRB, MDM2, NGF, FGFR2, CSF3, CSF2, FGFR1, PRKCZ, CCL2, FGFR3, TNF, ERBB4, CSF1, ERBB2, SOX2, KITLG, BCL2L1, KIT, ITGB1, TIMP1, IGF1R, KRAS, INS, NPM1, CD24, EGF, RUNX2, IL4, BMP4, IL6, FLT1, IL7, FLT3, IGF1, IGF2, STAT1, KDR, NRAS, CDKN1A, NOTCH1, CDKN1B, HDAC2, HDAC1, JAK2, MTOR, IL2 | 7.31E-52 |
| Positive regulation of macromolecule biosynthetic process | 97 | 2.236569 | 4.32E-54 | E2F1, HRAS, CDX2, PDGFB, XRCC6, TLR1, STAT5B, PPARG, FOXO1, TLR3, TLR4, NFKB1, ZEB1, FOXO3, TLR7, SHH, TGFB1, IL10, TLR8, GLI1, CTNNB1, TLR9, AKT1, WNT1, FOS, CCNE1, HMOX1, IFNG, IL1B, SHC1, YAP1, CCNA2, MYC, FGF2, AKT2, EGR1, AR, RELA, TP53, LEF1, RB1, CDK4, IRS1, CDK2, MAPK1, CD86, EP300, HIF1A, HNF4A, CD80, JUN, F2, VEGFA, PDGFRA, SMARCA4, CSF2, TNF, SOX2, KITLG, NFKBIA, SOX9, TCF7L2, WT1, STAT6, IGF1R, TNFRSF1A, IL17A, INS, SMARCB1, THBS1, RUNX2, BMP4, IL4, IL6, BMP2, CEBPB, CREB1, CREBBP, SMAD4, IGF1, SMAD3, IGF2, SMAD2, KAT5, FOXP3, STAT3, BRCA1, NOTCH1, HDAC2, SP1, HDAC1, ETS1, MAPK14, IRF1, MTOR, ENG, KLF4, IL2 | 7.87E-51 |

BP: biological process, FDR: False discovery rate

S7 Table: TOP10-Gene ontology-CC of TOP300 target genes of colorectal cancer

| Term | Count | % | P Value | Genes | FDR |
| --- | --- | --- | --- | --- | --- |
| Nucleoplasm | 72 | 1.660134 | 2.81E-23 | XRCC5, E2F1, CDX2, XRCC6, EZH2, FOXO1, NFKB1, ZEB1, CTNNB1, AKT1, FOS, CCNE1, CASP3, CDKN2A, H2AFX, YAP1, TOP2A, CCNA2, MYC, TERT, CDK1, RELA, POLE, TP53, LEF1, RB1, CDK4, CDK2, RAD51, MAPK1, CCND1, EP300, HIF1A, HNF4A, JUN, MAPK3, MDM2, MAPK8, HMGB1, MCL1, SOX2, MGMT, CHEK1, CHEK2, TCF7L2, WT1, SMARCB1, NPM1, RUNX2, ERCC1, ACTB, CREB1, CREBBP, SMAD4, SMAD3, BRCA2, SMAD2, ATR, KAT5, SIRT1, ATM, BRCA1, CCNB1, CDKN1A, HDAC2, HDAC1, PLK1, MAPK14, POLD1, PCNA, PARP1, KLF4 | 3.87E-20 |
| Organelle lumen | 102 | 2.351856 | 3.78E-21 | XRCC5, CDX2, PTGS2, PDGFB, XRCC6, FOXO1, TGFB1, CTNNB1, CDKN2A, H2AFX, CCNA2, RELA, POLE, MAPK1, VEGFC, HIF1A, EP300, HNF4A, JUN, VEGFA, MAPK3, MAPK8, NGF, HMGB1, MCL1, SOX2, CHEK1, CHEK2, SOX9, WT1, TIMP1, SMARCB1, ALB, INS, NPM1, EGF, RUNX2, FN1, MKI67, CREBBP, SMAD4, SMAD3, BRCA2, SMAD2, ATR, HGF, KAT5, BRCA1, ATM, CDKN1A, HDAC2, HDAC1, PLK1, POLD1, PCNA, PARP1, ABL1, KLF4, E2F1, EZH2, NFKB1, ZEB1, AKT1, FOS, CCNE1, CASP3, HMOX1, CASP8, NOS3, YAP1, MYC, TOP2A, TERT, CDK1, CYCS, TP53, LEF1, RB1, CDK4, CDK2, RAD51, CCND1, MDM2, MGMT, KIT, TCF7L2, STAT6, HSPA5, THBS1, ERCC1, ACTB, CEBPB, CREB1, IGF1, IGF2, STAT1, SIRT1, STAT3, SOD2, CCNB1, SP1, MAPK14, JAK2 | 5.20E-18 |
| Membrane-enclosed lumen | 103 | 2.374914 | 4.52E-21 | XRCC5, CDX2, PTGS2, PDGFB, XRCC6, FOXO1, TGFB1, CTNNB1, CDKN2A, H2AFX, CCNA2, RELA, POLE, MAPK1, VEGFC, HIF1A, EP300, HNF4A, JUN, VEGFA, MAPK3, MAPK8, NGF, HMGB1, MCL1, SOX2, CHEK1, CHEK2, SOX9, WT1, TIMP1, SMARCB1, ALB, INS, NPM1, EGF, RUNX2, FN1, MKI67, CREBBP, SMAD4, SMAD3, BRCA2, SMAD2, ATR, HGF, KAT5, BRCA1, ATM, CDKN1A, HDAC2, HDAC1, PLK1, POLD1, PCNA, PARP1, ABL1, KLF4, E2F1, EZH2, NFKB1, ZEB1, AKT1, FOS, CCNE1, CASP3, HMOX1, CASP8, NOS3, CAT, YAP1, MYC, TOP2A, TERT, CDK1, CYCS, TP53, LEF1, RB1, CDK4, CDK2, RAD51, CCND1, MDM2, MGMT, KIT, TCF7L2, STAT6, HSPA5, THBS1, ERCC1, ACTB, CEBPB, CREB1, IGF1, IGF2, STAT1, SIRT1, STAT3, SOD2, CCNB1, SP1, MAPK14, JAK2 | 6.22E-18 |
| Extracellular space | 59 | 1.360387 | 5.71E-20 | MMP9, WNT3A, CRP, MMP7, IL13, FASLG, IL15, MMP3, CXCL12, MMP2, SHH, TGFB1, IL10, APOE, HMOX1, IFNG, IL1B, FGF2, EGFR, ICAM1, VEGFC, CD40LG, F2, VEGFA, NGF, CSF3, CXCL1, WNT5A, CSF2, CCL2, TNF, ERBB3, CSF1, KITLG, KIT, IL17A, INS, ALB, EGF, THBS1, SPP1, FN1, BMP4, IL4, IL6, BMP2, FLT1, IL7, IGF1, IGF2, ADIPOQ, PLG, PROM1, LEP, AFP, PECAM1, MPO, ENG, SELE, IL2 | 7.85E-17 |
| Cytosol | 81 | 1.86765 | 2.12E-18 | PPARG, STAT5B, FOXO1, RPS6KB1, NFKB1, FOXO3, PTEN, CTNNB1, WNT2, AKT1, ACTG1, CDC42, CCNE1, CASP3, CASP9, HMOX1, CASP8, PIK3CA, SHC1, PRKACA, NOS3, CAT, FAS, AKT2, PRKCA, PPP2R1A, CDK1, HSP90AA1, PIK3CB, RELA, CYCS, TP53, CDK4, PRKCD, BCL2L11, CDK2, MAPK1, CCND1, JUN, LCK, MAPK3, MDM2, MAPK8, PRKCZ, CAV1, XIAP, GRB2, NFKBIA, BCL2L1, TCF7L2, SRC, TYMS, PTK2, SOS1, NPM1, RAC1, IDH1, HSPA5, HSPA8, PIK3R1, AXIN1, PIK3R2, ACTB, MAP2K1, CBL, SMAD4, SMAD3, RAF1, SMAD2, PTPN11, CCNB1, CDKN1A, CDKN1B, HDAC1, PLK1, MAPK14, GSK3B, TSC2, JAK2, MTOR, ABL1 | 2.91E-15 |
| Nuclear lumen | 85 | 1.95988 | 2.27E-18 | XRCC5, E2F1, CDX2, EZH2, XRCC6, FOXO1, NFKB1, ZEB1, CTNNB1, AKT1, FOS, CCNE1, CASP3, CDKN2A, HMOX1, CASP8, H2AFX, NOS3, YAP1, MYC, TOP2A, CCNA2, TERT, CDK1, RELA, POLE, TP53, LEF1, RB1, CDK4, CDK2, RAD51, MAPK1, CCND1, EP300, HIF1A, HNF4A, JUN, MAPK3, MDM2, MAPK8, HMGB1, MCL1, SOX2, MGMT, CHEK1, KIT, CHEK2, SOX9, TCF7L2, WT1, STAT6, SMARCB1, NPM1, RUNX2, ERCC1, ACTB, CEBPB, MKI67, CREB1, CREBBP, SMAD4, SMAD3, BRCA2, SMAD2, ATR, STAT1, KAT5, SIRT1, STAT3, ATM, BRCA1, CCNB1, CDKN1A, HDAC2, HDAC1, SP1, PLK1, MAPK14, POLD1, PCNA, JAK2, PARP1, ABL1, KLF4 | 3.12E-15 |
| Extracellular region part | 67 | 1.544847 | 5.84E-18 | WNT3A, MMP9, CRP, MMP7, IL13, FASLG, IL15, MMP3, CXCL12, MMP2, MMP1, IL10, SHH, TGFB1, WNT2, WNT1, CD44, APOE, HMOX1, IFNG, IL1B, FGF2, EGFR, ICAM1, MMP14, VEGFC, CD40LG, F2, VEGFA, NGF, CSF3, CXCL1, WNT5A, CSF2, CCL2, TNF, ERBB3, CSF1, KITLG, KIT, TIMP1, IL17A, ALB, INS, EGF, THBS1, SPP1, FN1, BMP4, IL4, IL6, BMP2, FLT1, LGALS3, IL7, IGF1, IGF2, ADIPOQ, PLG, PROM1, LEP, AFP, VWF, PECAM1, MPO, ENG, SELE, IL2 | 8.04E-15 |
| Intracellular organelle lumen | 92 | 2.121282 | 2.77E-16 | XRCC5, E2F1, CDX2, PTGS2, EZH2, XRCC6, FOXO1, NFKB1, ZEB1, TGFB1, CTNNB1, AKT1, CCNE1, FOS, CASP3, CDKN2A, HMOX1, CASP8, H2AFX, NOS3, YAP1, MYC, TOP2A, CCNA2, TERT, CDK1, RELA, CYCS, POLE, TP53, LEF1, RB1, CDK4, CDK2, RAD51, MAPK1, CCND1, EP300, HIF1A, HNF4A, JUN, MAPK3, MDM2, MAPK8, NGF, HMGB1, MCL1, SOX2, MGMT, CHEK1, KIT, CHEK2, SOX9, TCF7L2, WT1, STAT6, SMARCB1, INS, NPM1, HSPA5, RUNX2, ERCC1, ACTB, CEBPB, MKI67, CREB1, CREBBP, SMAD4, SMAD3, BRCA2, SMAD2, IGF2, ATR, STAT1, KAT5, SIRT1, STAT3, ATM, BRCA1, SOD2, CCNB1, CDKN1A, HDAC2, HDAC1, SP1, PLK1, MAPK14, POLD1, PCNA, JAK2, PARP1, ABL1, KLF4 | 3.11E-13 |
| Membrane raft | 24 | 0.553378 | 3.98E-14 | PRKCA, PRKCZ, CAV1, TNF, ERBB4, PTGS2, ERBB2, CBL, FASLG, TLR4, IRS1, SRC, SHH, WNT2, TNFRSF1A, KRAS, HMOX1, LCK, TSC2, CASP8, NOS3, JAK2, CD24, SELE | 5.48E-11 |
| Cell surface | 35 | 0.807009 | 1.19E-13 | FGFR2, CAV1, TNF, PDGFB, TLR2, FASLG, RPS6KB1, TLR4, ITGB3, KIT, ITGB1, TGFB1, SHH, CD44, APOE, HSPA5, FAS, CD24, THBS1, HSPA8, ICAM1, SELL, FLT3, CTLA4, CD40, PROM1, NCAM1, VWF, CD86, CD80, CD34, VEGFA, CD274, HSPB1, ENG | 1.64E-10 |

CC: cell component, FDR: False discovery rate

S8 Table: TOP10-KEGG of TOP300 target genes of colorectal cancer

| Term | Count | % | P Value | Genes | FDR |
| --- | --- | --- | --- | --- | --- |
| Pathways in cancer | 121 | 2.789947 | 2.08E-82 | HRAS, PTGS2, PDGFB, WNT3A, MMP9, STAT5B, FOXO1, MMP2, MMP1, TGFB1, SHH, CTNNB1, WNT2, WNT1, CDKN2A, PIK3CA, FAS, EGFR, RET, PIK3CB, RELA, MAPK1, VEGFC, EP300, HIF1A, JUN, VEGFA, MAPK3, PDGFRA, MAPK9, PDGFRB, MAPK8, FGFR2, WNT5A, FGFR1, FGFR3, GRB2, ERBB2, NFKBIA, BCL2L1, ITGB1, PTK2, KRAS, RAC1, EGF, AXIN2, FN1, CSF1R, AXIN1, BMP4, BMP2, MAP2K1, MET, CREBBP, SMAD4, BRCA2, SMAD3, SMAD2, HGF, CDKN1A, CDKN1B, HDAC2, HDAC1, ETS1, MTOR, ABL1, E2F1, FGF8, PPARG, MLH1, FASLG, NFKB1, PTEN, GLI1, AKT1, CDC42, FOS, CCNE1, CASP3, CASP9, CASP8, RHOA, FGF2, MYC, AKT3, AKT2, PRKCA, AR, HSP90AA1, CYCS, TP53, LEF1, CDK6, RB1, CDK4, CDK2, RAD51, CCND1, MDM2, XIAP, KITLG, CDH1, KIT, TCF7L2, IGF1R, SOS1, PIK3R1, PIK3R2, MSH6, IL6, FLT3, MSH2, CBL, IGF1, RAF1, MAPK10, STAT1, STAT3, NRAS, GSK3B, JAK1 | 2.37E-79 |
| Prostate cancer | 52 | 1.198985 | 4.39E-45 | E2F1, HRAS, PDGFB, FOXO1, NFKB1, PTEN, CTNNB1, AKT1, CCNE1, CASP9, PIK3CA, AKT3, AKT2, EGFR, AR, HSP90AA1, PIK3CB, RELA, TP53, LEF1, RB1, CDK2, MAPK1, CCND1, EP300, MAPK3, PDGFRA, MDM2, PDGFRB, FGFR2, FGFR1, GRB2, ERBB2, NFKBIA, TCF7L2, IGF1R, KRAS, INS, SOS1, EGF, PIK3R1, PIK3R2, MAP2K1, CREB1, CREBBP, IGF1, RAF1, IGF2, NRAS, CDKN1A, CDKN1B, GSK3B, MTOR | 5.01E-42 |
| Pancreatic cancer | 42 | 0.968411 | 4.96E-36 | E2F1, ERBB2, NFKB1, BCL2L1, TGFB1, AKT1, CDC42, KRAS, CDKN2A, CASP9, RAC1, PIK3CA, EGF, AKT3, PIK3R1, PIK3R2, AKT2, EGFR, MAP2K1, PIK3CB, RELA, SMAD4, TP53, SMAD3, RAF1, BRCA2, CDK6, SMAD2, RB1, MAPK10, STAT1, CDK4, STAT3, RAD51, MAPK1, VEGFC, CCND1, VEGFA, MAPK3, JAK1, MAPK9, MAPK8 | 5.66E-33 |
| Colorectal cancer | 44 | 1.014526 | 4.15E-35 | GRB2, MLH1, TCF7L2, TGFB1, CTNNB1, AKT1, IGF1R, FOS, CASP3, KRAS, CASP9, SOS1, RAC1, PIK3CA, AXIN2, MYC, AKT3, PIK3R1, AXIN1, PIK3R2, AKT2, EGFR, MSH6, MAP2K1, MSH2, PIK3CB, MET, CYCS, SMAD4, TP53, SMAD3, LEF1, RAF1, SMAD2, MAPK10, MAPK1, CCND1, GSK3B, JUN, MAPK3, PDGFRA, PDGFRB, MAPK9, MAPK8 | 4.73E-32 |
| Chronic myeloid leukemia | 41 | 0.945354 | 1.32E-33 | E2F1, HRAS, GRB2, STAT5B, NFKBIA, NFKB1, BCL2L1, TGFB1, AKT1, KRAS, CDKN2A, SOS1, PIK3CA, SHC1, MYC, AKT3, PIK3R1, PIK3R2, AKT2, MAP2K1, PIK3CB, RELA, CBL, SMAD4, TP53, SMAD3, RAF1, CDK6, RB1, CDK4, PTPN11, MAPK1, NRAS, CDKN1A, CCND1, CDKN1B, HDAC2, HDAC1, MAPK3, MDM2, ABL1 | 1.51E-30 |
| Glioma | 36 | 0.830067 | 2.77E-30 | E2F1, HRAS, PDGFB, GRB2, PTEN, AKT1, IGF1R, KRAS, CDKN2A, SOS1, PIK3CA, SHC1, EGF, AKT3, PIK3R1, PIK3R2, AKT2, EGFR, PRKCA, MAP2K1, PIK3CB, TP53, RAF1, IGF1, CDK6, RB1, CDK4, MAPK1, NRAS, CDKN1A, CCND1, MAPK3, PDGFRA, MDM2, PDGFRB, MTOR | 3.16E-27 |
| Endometrial cancer | 33 | 0.760895 | 9.81E-30 | HRAS, GRB2, ERBB2, MLH1, CDH1, FOXO3, TCF7L2, PTEN, CTNNB1, AKT1, KRAS, CASP9, SOS1, PIK3CA, EGF, AXIN2, MYC, AKT3, PIK3R1, AXIN1, PIK3R2, AKT2, EGFR, MAP2K1, PIK3CB, TP53, LEF1, RAF1, MAPK1, NRAS, CCND1, GSK3B, MAPK3 | 1.12E-26 |
| Melanoma | 37 | 0.853124 | 3.07E-29 | E2F1, FGFR1, HRAS, FGF8, PDGFB, CDH1, PTEN, AKT1, IGF1R, KRAS, CDKN2A, PIK3CA, EGF, FGF2, AKT3, PIK3R1, PIK3R2, AKT2, EGFR, MAP2K1, PIK3CB, MET, TP53, IGF1, RAF1, CDK6, RB1, HGF, CDK4, MAPK1, NRAS, CDKN1A, CCND1, MAPK3, PDGFRA, MDM2, PDGFRB | 3.50E-26 |
| ErbB signaling pathway | 39 | 0.899239 | 9.10E-28 | HRAS, ERBB4, GRB2, ERBB3, ERBB2, STAT5B, RPS6KB1, SRC, AKT1, PTK2, KRAS, SOS1, PIK3CA, SHC1, EGF, MAP2K7, MYC, AKT3, PIK3R1, PIK3R2, AKT2, EGFR, PRKCA, MAP2K1, PIK3CB, CBL, RAF1, MAPK10, MAPK1, NRAS, CDKN1A, CDKN1B, GSK3B, JUN, MAPK3, MAPK9, MAPK8, MTOR, ABL1 | 1.04E-24 |
| Focal adhesion | 55 | 1.268158 | 6.47E-27 | HRAS, PDGFB, PTEN, CTNNB1, AKT1, ACTG1, CDC42, RHOA, PIK3CA, SHC1, AKT3, AKT2, PRKCA, EGFR, PIK3CB, MAPK1, VEGFC, CCND1, CCND2, JUN, MAPK3, VEGFA, PDGFRA, PDGFRB, MAPK9, MAPK8, CAV1, XIAP, GRB2, ERBB2, ITGB3, ITGB1, SRC, PXN, IGF1R, PTK2, SOS1, RAC1, EGF, THBS1, PIK3R1, PIK3R2, FN1, SPP1, ACTB, FLT1, MAP2K1, MET, IGF1, RAF1, MAPK10, HGF, KDR, VWF, GSK3B | 7.38E-24 |

KEGG: Kyoto Encyclopedia of Genes and Genomes, FDR: False discovery rate


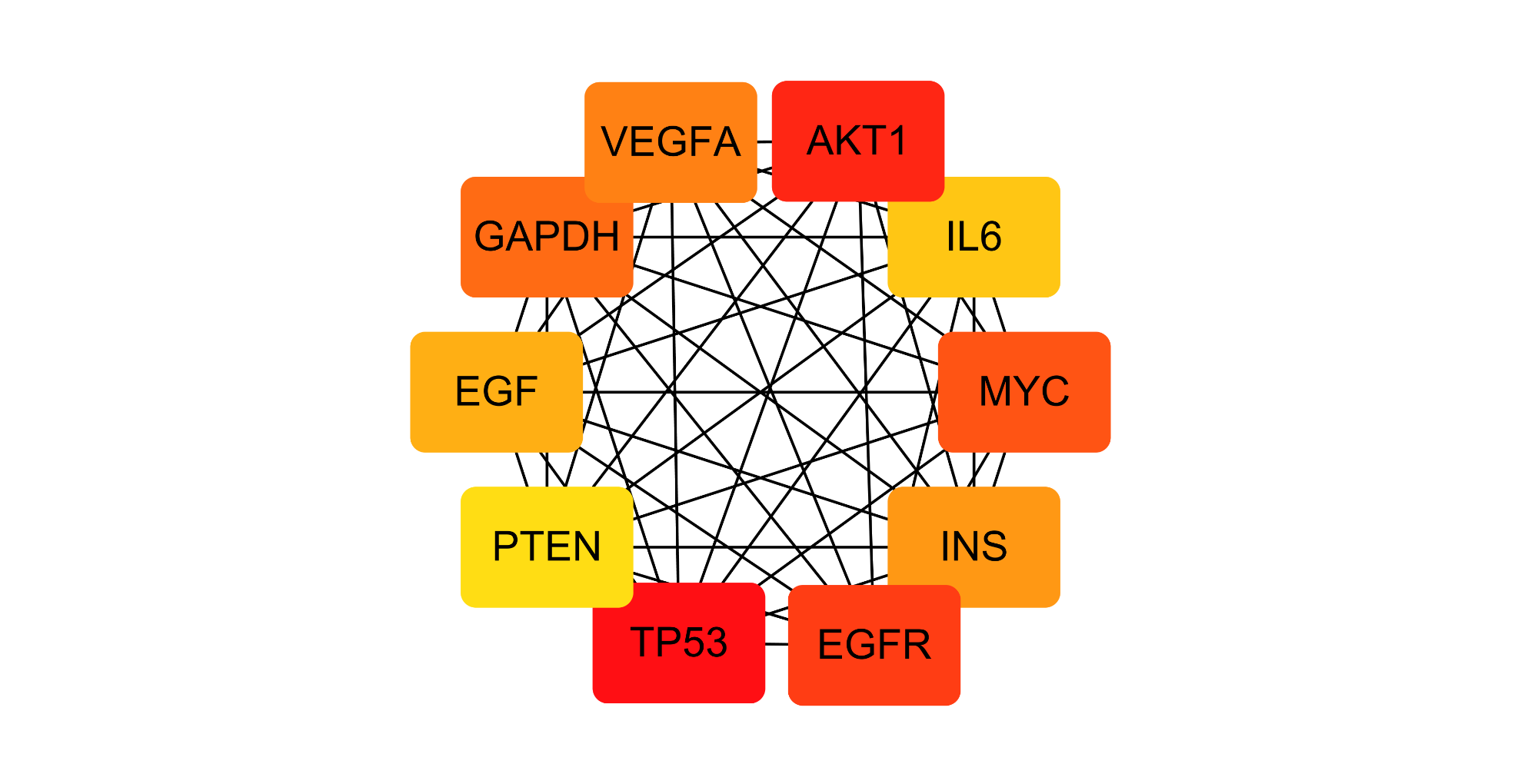


S1 Fig: the PPI network of colorectal cancer related TOP10-hub genes
